# Early versus Delayed Surgery in Patients with Left-Sided Infective Endocarditis and Stroke

**DOI:** 10.3390/jcdd10080356

**Published:** 2023-08-21

**Authors:** Jamila Kremer, Joshua Jahn, Sabrina Klein, Mina Farag, Tobias Borst, Matthias Karck

**Affiliations:** 1 Department of Cardiac Surgery, Heidelberg University Hospital, Im Neuenheimer Feld 420, 69120 Heidelberg, Germany; 2 Department of Infectious Diseases, Medical Microbiology and Hygiene, Heidelberg University Hospital, Im Neuenheimer Feld 324, 69120 Heidelberg, Germany; 3 Pharmacy Department, Erlangen University Hospital, Palmsanlage 3, 91054 Erlangen, Germany

**Keywords:** infective endocarditis (IE), stroke, timing of surgery, valve surgery, neurological complications, cerebral embolism, intracranial haemorrhage, heart failure, aortic valve

## Abstract

Background: Timing of surgery remains controversial in patients with infective endocarditis and stroke. Guidelines on infective endocarditis suggest delaying surgery for up to 4 weeks. However, with early heart failure due to progression of the infection or recurrent septic embolism, urgent surgery becomes imperative. Methods: Out of 688 patients who were surgically treated for left-sided infective endocarditis, 187 presented with preoperative neurological events. The date of cerebral stroke onset was documented in 147 patients. The patients were stratified according to timing of surgery: 61 in the early group (0–7 days) vs. 86 in the delayed group (>7 days). Postoperative neurological outcome was assessed by the modified Rankin Scale. Results: Preoperative sepsis was more prevalent in patients with preoperative neurological complications (46.0% vs. 29.5%, *p* < 0.001). Patients with haemorrhagic stroke were operated on later (19.8% vs. 3.3%, *p =* 0.003). Postoperative cerebrovascular accidents were comparable between both groups (*p* = 0.13). Overall, we observed good neurological outcomes (*p* = 0.80) and a high recovery rate, with only 5% of cases showing neurological deterioration after surgery (*p* = 0.29). In-hospital mortality and long-term survival were not significantly different in the early and delayed surgery groups (log-rank, *p* = 0.22). Conclusions: Early valve surgery in high-risk patients with infective endocarditis and stroke can be performed safely and is not associated with worse outcomes.

## 1. Introduction

In patients with infective endocarditis (IE), neurological sequalae occur in 20–40% of cases and are the second most prevalent complication with poorer clinical outcome [1,2,3,4]. The timing of surgery remains controversial in patients with active IE and cerebral stroke (CS) and should be carefully evaluated by a multidisciplinary team [5]. Full heparinization for cardiopulmonary bypass (CPB) increases the risk of secondary bleeding or extension of cerebral haemorrhages. Continuous flow during CPB with hypotension can enhance cerebral oedema with a disrupted blood-brain barrier [6,7,8]. The guidelines on IE management are somewhat ambiguous; they suggest delaying surgery for up to 4 weeks [9]. However, with imminent heart failure, progression of the infection or recurrent septic embolism, urgent surgery is imperative. While earlier studies favoured delaying surgery due to high morbidity and mortality, in the recent literature, growing evidence in favour of early surgery shows that the risk of neurological deterioration in patients with IE and stroke, particularly in non-haemorrhagic infarction, is low [3,6,10,11,12,13,14,15,16].

In this retrospective study, we assessed the rate of perioperative CS in patients with left-sided IE and variables determining the timing of cardiac valve surgery after ischemic or haemorrhagic CS, also focusing on patients’ neurological outcomes.

## 2. Materials and Methods

### 2.1. Ethics Statement

The study was conducted according to the Declaration of Helsinki and was approved by the ethics committee of the University of Heidelberg, S-424/2020. Written informed consent was waived due to the retrospective nature of the study and the anonymization of the data.

### 2.2. Patients

**Figure 1:**
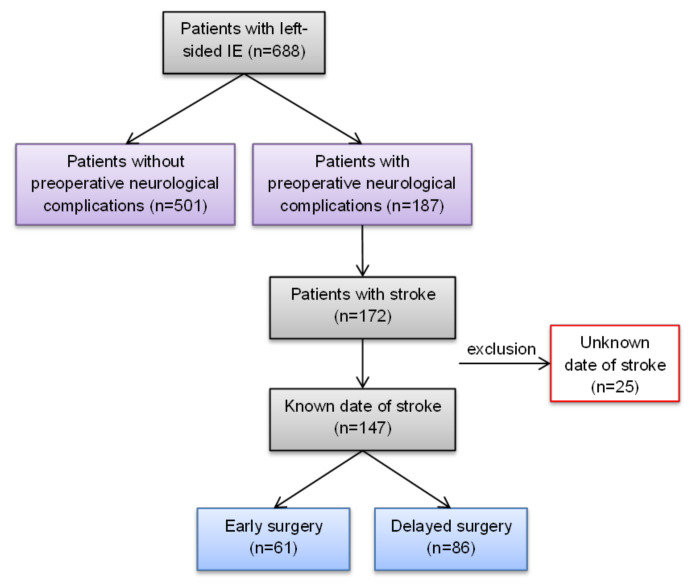
Flow chart of patient selection. IE: infective endocarditis.

Patient data were collected from our institutional database from 688 consecutive patients with left-sided IE operated on between 1998 and 2018. Out of the 688 patients, 187 patients (27.2%) had ≥1 preoperative neurological complication. To allow for differentiation of timing of surgery in patients with a neurological complication related to left-sided IE, only patients with the known date of stroke, in total 147 patients (21.4%), were allocated according to their timing of surgery: 61 patients to the “early surgery group” (≤7 days), and 86 patients to the “delayed surgery group” (>7 days). Differentiation of ischemic or haemorrhagic CS and the location of the stroke area were obtained through preoperative imaging, either cerebral computed tomography (CT) or magnetic resonance imaging (MRI). A flow chart of patient selection is depicted in Figure 1.

### 2.3. Endocarditis Diagnosis and Surgery

Infective endocarditis was diagnosed by the modified Duke criteria [17], and vegetations were classified by transthoracic or transoesophageal echocardiography. Microbiological information was obtained from blood cultures and intraoperative heart tissue specimens.

Cardiopulmonary bypass was performed under mild hypothermia (32–34 °C) and full heparinization (activated clotting time > 480 s). As a tool to predict in-hospital mortality in patients with active left-sided infective endocarditis undergoing cardiac surgery, the RISK-E score was used. This score uses standard heart surgery variables as well as IE-specific factors [18].

Follow-up time for survival was defined as the time after surgery to either patient death or last alive patient contact.

### 2.4. Neurological Complications

Preoperative neurological complications included stroke, transient ischemic attack (TIA), septic encephalopathy/meningitis and brain abscess. Stroke was defined as an acute neurological deficit lasting >24 h. Haemorrhage included primary intracerebral haemorrhage, haemorrhagic infarction, and subarachnoid haemorrhage. Primary haemorrhage was defined as intracerebral bleeding without signs of former ischemic stroke. Secondary haemorrhage was noted as haemorrhagic conversion of an ischemic stroke. Neurological symptoms were classified as focal symptoms (hemiparesis, diplopia, aphasia) or non-focal symptoms (headache, altered mental status).

### 2.5. Postoperative Neurological Outcome

New cerebrovascular accidents (stroke, TIA and seizure) and the rate of haemorrhagic transformation of preoperative IE-related strokes were analysed. Postoperative brain imaging was not routinely performed in all patients, just in cases of new neurological symptoms or after neurologist consultation due to unclear postoperative neurological status.

For postoperative neurological evaluation, we used the modified Rankin Scale (mRS) which was gathered during in-hospital stay. In 123 patients, an assessment of the postoperative neurological status via mRS was possible. The reasons for missing mRS were insufficient data, death during surgery, or patients’ transfer to another intensive care unit/hospital without prior neurological examination. Favourable neurological outcome was defined as a postoperative mRS of 0–1.

### 2.6. Statistics

Categorical variables were reported as frequencies and percentages and were analysed by Pearson’s chi-square test or Fisher’s exact test when the minimum expected cell count was <5. Continuous variables were compared by the Mann–Whitney U test and were reported as medians and 25th and 75th percentiles. Survival was calculated using the Kaplan–Meier method. Groups were compared using the log-rank test. Risk factors for postoperative neurological complications were identified using a multivariable logistic regression analysis. After univariable analysis, all variables with a *p* value < 0.10 were included in the multivariable analysis using a forward stepwise selection (*p_in_* = 0.05, *p_out_* = 0.10). The results are presented as an odds ratio (OR) with a corresponding 95% confidence interval (CI) and *p* value. All the tests were performed 2-sided, and a *p* value < 0.05 was considered statistically significant. All statistical analyses were performed using the IBM SPSS Statistics version 25 software (SPSS, Chicago, IL, USA).

## 3. Results

### 3.1. Patient Selection

Out of 688 patients with left-sided IE undergoing heart valve surgery between 1998 and 2018, 187 patients (27.2%) had ≥1 preoperative neurological complication, with the majority of the 150 patients (80.2%) suffering from an ischemic CS. Non-ischemic events included 22 (11.8%) haemorrhagic CS, 5 (2.7%) TIAs, 21 (11.2%) septic encephalopathies/meningitides cases and 4 patients (2.1%) with a brain abscess. The clinical evaluation recorded 114 patients (61.0%) with neurological symptoms, 89 (47.6%) having focal symptoms and 25 (13.4%) non-focal. The median modified Rankin Scale was 2 (1–3).

### 3.2. Pathology of Endocarditis

A total of 557 (81.0%) patients were in New York Heart Association (NYHA) class III (n = 321, 46.7%) or IV (n = 236, 34.3%) at the time of surgery. The mitral valve was significantly more frequently affected in patients with preoperative neurological events (62.6% vs. 51.9%, *p* = 0.01). Preoperative sepsis was more prevalent in the latter (46.0% vs. 29.5%, *p* < 0.001). The median vegetation size was 15 mm (10–20 mm). There was a significant intergroup difference, with larger vegetations in patients with neurological events (18 mm vs. 13 mm, *p* < 0.001).

*Staphylococcus aureus* was the most common pathogen isolated (21.9%), being more prominent in patients with preoperative neurological sequalae (33.2% vs. 17.8%, *p* < 0.001), whereas patients without these complications were more likely to have *Enterococcus* spp. IE (14.6% vs. 7.0%, *p* = 0.007). A detailed description is listed in Supplementary Material, Table S1.

### 3.3. Early vs. Delayed Surgery after Preoperative Stroke

For the “early surgery” vs. “delayed surgery” analysis, 147 patients (21.4%) with preoperative stroke and known onset of CS were included (Figure 1). The age and gender distributions were comparable between both groups (median age: 64 (57–72) vs. 64 (55–71) years, *p* = 0.85; female patients: 27.9% vs. 34.9%, *p* = 0.37). The RISK-E score was 26 in the early surgery group compared with 24 in the delayed surgery group, which translates to a predicted in-hospital mortality of 32.8% vs. 28.9%, *p* = 0.39.

Early surgery was chosen as a surgical strategy in patients with higher C-reactive protein, annular abscess, and larger vegetation, with significance levels of *p* = 0.01, *p* = 0.02 and *p* = 0.006, respectively. Patients with NYHA class IV were numerically more frequent in the early surgery group (49.2% vs. 33.7%, *p* = 0.06). *Staphylococcus aureus* (32.7%) was the most common pathogen isolated. The infective endocarditis characteristics of the early and the delayed surgery groups are found in Table 1.

#### 3.3.1. Neurological Status

Patients with a haemorrhagic CS were more often in the delayed group (19.8% vs. 3.3%, *p* = 0.003). Patients with severe stroke according to the mRS were operated on later (*p* = 0.03). In most patients (60.5%), cerebral lesions affected multiple different brain areas. The middle cerebral artery (18.4%) was the single most common location (Table 2).

#### 3.3.2. Perioperative and Postoperative Outcome

The median duration from onset of CS to surgery was 3 days (1–6 days) in the early and 22 days (13–26 days) in the delayed group. Mechanical prosthesis was the preferred type of valve replacement used in the early group (59.0% vs. 41.9%, *p* = 0.04) (Supplementary Material, Table S2).

Postoperative sepsis was more frequent in patients operated on earlier (49.2% vs. 26.7%, *p* = 0.005), with no significant difference in other postoperative outcome variables (Table 3).

Regarding postoperative neurological outcomes, no significant difference in new cerebrovascular accidents was found (*p*= 0.13). Three patients had a postoperative stroke, two in the early group and one in the delayed group. The first patient out of the early surgery group was a 67-year-old male who experienced an ischemic stroke on his first postoperative day after receiving a mechanical aortic valve replacement because of prosthetic valve IE. At the end of his intensive care unit stay, he showed no neurological symptoms or residuals deficits. The second case was a 71-year-old male patient who initially had a cerebral haemorrhage secondary to an embolic lesion before surgery. The postoperative control CTs showed a large right-sided media infarction/haemorrhagic transformation with a perifocal oedema and a slight midline shift. The patient received a trepanation and haematoma evacuation and later died on the same day.

A 79-year-old male in the delayed surgery group had a right-sided partial media infarction on his first postoperative day after having a biological aortic valve replacement due to prosthetic valve IE. He showed signs of left-sided hemiplegia and hemineglect. He partially recovered but had some neurological impairments at his discharge.

There was one TIA in the early group; this patient’s neurological symptoms resolved spontaneously within a few hours. Overall, three patients experienced seizures, which were all successfully medically treated.

There was no significant difference in the median postoperative mRS between both groups (*p*= 0.80). There were 39 patients (63.9%) in the early and 52 patients (60.5%) in the delayed group who showed a favourable neurological outcome, *p*= 0.25.

**Figure 2:**
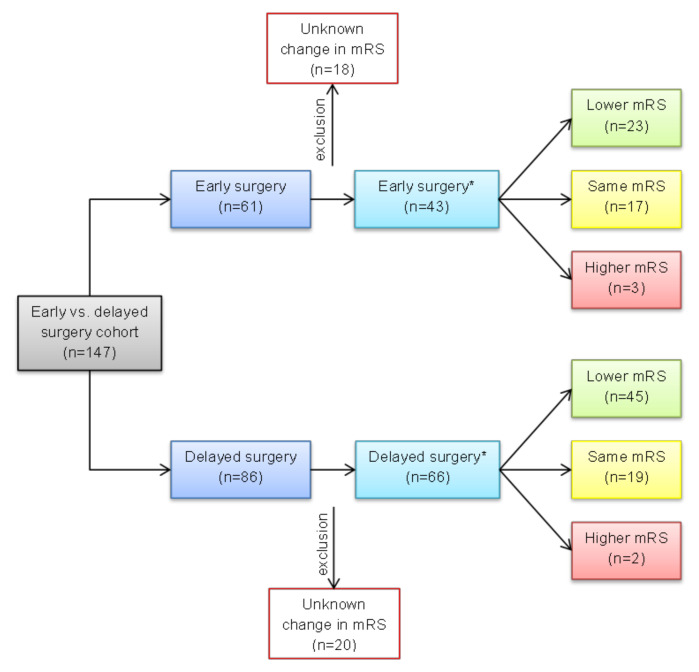
Neurological recovery in early vs. delayed surgery groups after preoperative stroke assessed by changes in pre- to postoperative mRS. mRS: modified Rankin Scale; *: available data (n = 109).

Neurological recovery is depicted in Figure 2. Overall, 68 patients had a lower documented mRS and favourable neurological development postoperatively: 23 patients in the early group vs. 45 patients in the delayed group. No changes in the mRS were observed in 36 cases. A higher mRS was documented in three patients in the early group and in two patients in the delayed surgery group. The neurological recovery did not show any significant difference (*p* = 0.29).

**Figure 3:**
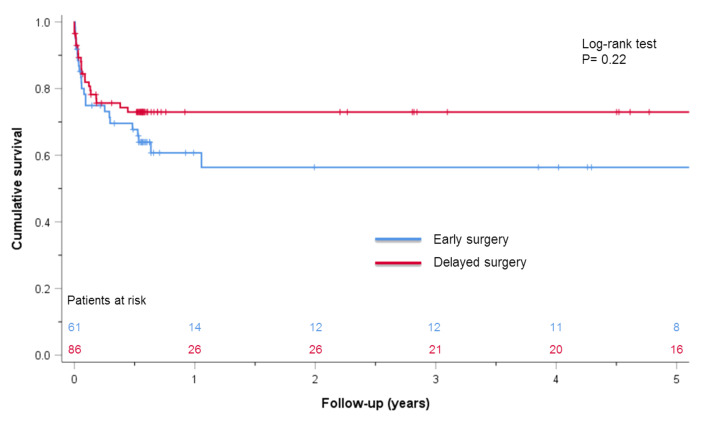
Kaplan–Meier survival analysis of early vs. delayed surgery after preoperative stroke. Early surgery (blue line) vs. delayed surgery (red line).

Left-sided IE with *Enterococcus* spp. (OR 7.533; 95% CI 1.184–47.924) and the duration of the operation (OR 1.059; 95% CI 1.002–1.119) were independent risk factors for postoperative neurological complications in patients with preoperative neurological sequalae, (Supplementary Material, Table S3). There was no significant difference in the in-hospital mortality (11.5% vs. 8.1%, *p* = 0.50) and long-term survival between early and delayed surgery groups (log-rank, *p* = 0.22) (Figure 3).

## 4. Discussion

The timing of heart surgery in patients with valvular infective endocarditis and acute stroke is crucial because neurological risks associated with systemic heparinization, as required for CPB, need to be weighed against a persisting threat of central embolization of valvular vegetation and decompensated heart failure [7]. Earlier reports indicated that the risk of haemorrhagic transformation of an embolic stroke is reduced when valve surgery is delayed, allowing for stabilization of the ischemic brain lesion. This view, however, was challenged by reports revealing no benefit in postponing surgery longer than 48 h from the onset of stroke [12,19].

### 4.1. Infective Valvular Endocarditis

Neurological complications occurred more often in patients with mitral valve IE compared to aortic valve IE (62.6% vs. 51.9%, *p* = 0.01). The median size of the vegetations in our cohort was 15 mm (10–20 mm). *Staphylococcus aureus* was the most common pathogen isolated, being more common in patients with preoperative neurological sequalae, whereas neurologically inapparent patients were more likely to be seropositive for *Enterococcus* spp. Interestingly, our subgroup analysis of all patients with documented preoperative CS showed that enterococcal IE was an independent risk factor for postoperative neurological complications. These results match data from Zhang et al. published recently [1]. Their group found that enterococcal IE was more common in patients with perioperative neurological complications as well.

Heart failure is the most common and most severe indication for surgery in IE patients, with almost two thirds of patients affected [5,20]. The risk of development of heart failure in untreated severe aortic regurgitation due to IE is high. In cases with an aortic valve endocarditis, heart failure is observed more often than in mitral valve IE cases [21,22]. Patients with heart failure require continuous and rigorous surveillance and evaluation of their clinical status. Delayed surgery entails the risk of rapid decompensation, which can worsen the outcome significantly [23,24]. Therefore, it is not surprising that heart failure is the most important predictor of early mortality in IE patients [5,22,25,26].

### 4.2. Timing of Surgery

Regarding the effect of timing of surgery, we could not observe a statistical difference in the in-hospital mortality (11.5% vs. 8.1%, *p* = 0.50) or long-term survival between patients operated on early or delayed after CS (log-rank, *p*= 0.22) in our retrospective study cohort. Furthermore, full heparinization for CPB in cases with documented preoperative stroke seemed not to have compromised postoperative haemorrhagic transformation of such preoperative strokes in our cohort [3,6,27].

Diab et al. reported that 6 out of 34 patients (17.6%) with preoperative intracranial haemorrhage developed a postoperative haemorrhagic infarction [28]. Patients operated on within one week had three-times more postoperative neurological events, in their analysis. This result contrasts with our findings which included only one postoperative neurological event in the entire cohort with preoperative haemorrhagic CS. Out of 19 patients with haemorrhagic CS, only one patient (5.2%) suffered from a postoperative secondary cerebral bleeding. The authors of the above-mentioned paper state that postoperative activated partial thromboplastin time was almost two-fold higher in their patients with postoperative intracranial haemorrhage [28]. As changes in perioperative coagulation status were not assessed in our study, the potential impact of this variable remains to be determined, especially in patients with mechanical valve replacement and preoperative IE-related CS.

When analysing clinical neurological status, there was no difference in the postoperative recovery status by mRS between early and delayed surgery in our study. In general, we observed good neurological outcomes, with 62% of cases showing favourable neurological rehabilitation during follow-up and only 5% of cases presenting neurological deterioration after surgery. These findings are comparable to the excellent results by Ruttmann et al. in their detailed study about neurological recovery in ischemic IE-related stroke [6].

The data of our study show that early surgery to prevent further clinical deterioration in patients with left-sided IE and neurological sequalae is feasible and entails minimal risk, which is comparable to the results from Piper et al. who performed early surgery within 72 h after CS, describing a low incidence of secondary haemorrhagic stroke [12]. Moreover, the time limit of 3 days as suggested in the latter study appears more aggressive compared with the respective recommendations of international guidelines [5,9]. Our results show no difference in mortality between early and delayed surgery; however, postoperative morbidity in relation to timing of surgery was not evaluated in this study. We only showed that the incidence of postoperative sepsis was significantly higher in patients within the early surgery group. The exacerbation of the clinical status of IE patients is reflected in an increased number of emergency procedures in the early group.

In another meta-analysis from Tam et al. “early surgery” (7 days vs. 14 days) in ischemic CS was compared with surgery after 21 and 28 days in patients with haemorrhagic CS. No difference was found in perioperative mortality and neurological deterioration in the ischemic CS group when surgery was performed within 7 days. However, in patients with haemorrhagic CS, the risk of perioperative mortality and neurological complications were found to be increased [15]. Only one patient in our cohort suffered from haemorrhagic transformation after CPB. Postoperative cerebrovascular accidents were comparable between both groups.

Taken together, our data underline the difficult conundrum in the case of IE treatment with neurological sequalae. Early surgery in patients in a deteriorating clinical state may worsen the surgical outcome. Yet, this risk needs to be weighed against the ongoing threat of left-sided IE, including uncontrolled heart failure, continued dissemination of the infection and the increase in embolic events. Pericàs et al. addressed these aspects in a prospective cohort study and found that *Staphylococcus aureus*, nosocomial acquisition, persistent bacteraemia, acute renal failure, central nervous system emboli and larger vegetation size were associated with higher rates of septic shock. They concluded that early identification of patients at risk for septic shock and timely surgical therapy are important corner stones to improve outcomes in IE patients [29]. In their 2016 published paper, Yanagawa et al. reviewed the literature, presented practical recommendations for clinicians in this special subset of patients and offered an algorithm for their management. They came to the same conclusion that more recent studies, as also seen in this paper, show no evidence of a worse outcome from earlier surgery [10].

At our institution, patients with left-sided IE and stroke are managed with a team approach. Endocarditis patients are discussed by an endocarditis board, which is a multidisciplinary meeting. This approach is recommended by the 2015 European Society of Cardiology guidelines for the management of infective endocarditis as it can reduce the 1-year mortality [5,30]. Various experts from different specialties discuss the type and duration of the antibiotic therapy and whether there is an indication for surgery. In patients with left-sided IE and stroke, particularly in cases with a large ischemic stroke or a haemorrhage, we do not have a standard or fixed approach. In fact, it requires, and probably will always require, a highly individual approach to evaluate the best possible treatment for every single patient in this challenging conundrum. In these cases, cerebral imaging is of utmost importance. A neurologist is consulted who carefully evaluates cerebral CT or MRI images for the potential of haemorrhagic transformation or aggravation of pre-existing intracranial bleeding. After the neurologist’s statement, each patient is discussed thoroughly in synopsis of all the medical findings and the clinical status of the patient. If an immediate surgery is required with high likelihood for a worsening of the cerebral findings, peri- and postoperative anticoagulation regimes are carefully managed to minimise the risk of bleeding aggravation.

The limitations of this study include the relatively small patient cohort as well as its retrospective nature in a single-centre study. Due to the small sample size, we did not perform a propensity score analysis. Neurological imaging in this study is missing data on the size of cerebral infarctions plus routine postoperative imaging to rule out silent events. Furthermore, morbidity following preoperative neurological complications needs to be addressed in future studies with a focus on anticoagulation in the postoperative setting.

## 5. Conclusions

Early valve surgery in high-risk patients with infective endocarditis and stroke can be performed safely and is not associated with a worse outcome, especially in patients with impaired cardiac function.

## Figures and Tables

**Table 1:** Infective endocarditis characteristics in early vs. delayed surgery groups after preoperative stroke.

Variable	Early Surgery (n = 61)	Delayed Surgery (n = 86)	*p*-Value
Previous IE	1 (1.6)	9 (10.5)	**0.046**
Affected valve			
Aortic	33 (54.1)	51 (59.3)	0.53
Mitral	42 (68.9)	50 (58.1)	0.19
Double valve	14 (23.0)	15 (17.4)	0.41
Prosthetic valve endocarditis	6 (9.8)	20 (23.3)	**0.04**
Sepsis	24 (39.3)	42 (48.8)	0.25
RISK-E score	26 (15–36)	24 (14–35)	0.39
Congestive heart failure	19 (31.1)	24 (27.9)	0.67
Embolic events	61 (100)	78 (90.7)	**0.02**
Brain	61 (100)	78 (90.7)	**0.02**
Peripheral emboli	19 (31.1)	17 (19.8)	0.11
Laboratory values			
C-reactive protein (mg/dL)	8.0 (4.2–18.9)	6.1 (2.5–9.9)	**0.01**
WBC (x10^9^/L)	9.5 (7.8–16.0)	10.0 (7.6–13.1)	0.49
Creatinine (mg/dL)	1.1 (0.8–1.9)	1.1 (0.7–1.7)	0.26
Bilirubin (mg/dL)	0.8 (0.5–3.0)	0.6 (0.3–0.9)	**0.02**
Causative microorganism			
Staphylococcus spp.	27 (44.3)	30 (34.9)	0.25
Staphylococcus aureus	22 (36.1)	26 (30.2)	0.46
MRSA	1 (1.6)	2 (2.3)	>0.99
Coagulase-negative staphylococci	5 (8.2)	4 (4.7)	0.49
Streptococcus spp.	15 (24.6)	21 (24.4)	0.98
Viridans group streptococci	8 (13.1)	9 (10.5)	0.62
Streptococcus bovis	4 (6.6)	9 (10.5)	0.41
Enterococcus spp.	3 (4.9)	6 (7.0)	0.74
Fungi	1 (1.6)	0	0.42
Other	5 (8.2)	4 (4.7)	0.49
Annular abscess	29 (47.5)	30 (34.9)	**0.02**
Vegetation	53 (86.9)	71 (82.6)	0.09
Vegetation size (mm)	20 (15–30)	16 (11–20)	**0.006**

Data are presented as n (%) or median (25th–75th percentile). Bold values indicate statistically significant differences (*p* < 0.05). IE: infective endocarditis; MRSA: methicillin-resistant *Staphylococcus aureus*; WBC: white blood cell.

**Table 2:** Neurological status of early vs. delayed surgery after preoperative stroke.

Variable	Early Surgery (n = 61)	Delayed Surgery (n = 86)	*p*-Value
Neurological symptoms	41 (67.2)	66 (76.7)	0.33
Focal	30 (49.2)	53 (61.6)	
Non-focal	11 (18.0)	13 (15.1)	
Modified Rankin Scale	1 (1–2)	2 (1-3)	**0.03**
Type of stroke			**0.003**
Ischemic stroke	59 (96.7)	69 (80.2)	
Haemorrhage	2 (3.3)	17 (19.8)	
Affected brain areas			0.64
Frontoparietal	3 (4.9)	7 (8.1)	
Middle cerebral artery	10 (16.4)	17 (19.8)	
Parietal	2 (3.3)	1 (1.2)	
Thalamus	1 (1.6)	2 (2.3)	
Occipital	2 (3.3)	0	
Brain stem	0	2 (2.3)	
Cerebellum	2 (3.3)	4 (4.7)	
Multiple sites	38 (62.3)	51 (59.3)	
Unknown	3 (4.9)	2 (2.3)	
Other neurological complications			
TIA	0	0	
Septic encephalopathy/meningitis	4 (6.6)	8 (9.3)	0.76
Brain abscess	2 (3.3)	1 (1.2)	0.57

Data are presented as n (%) or median (25th–75th percentile). Bold values indicate statistically significant differences (*p* < 0.05). TIA: transient ischemic attack.

**Table 3:** Postoperative outcome data of early vs. delayed surgery after preoperative stroke.

Variable	Early Surgery (n = 61)	Delayed Surgery (n = 86)	*p*-Value
Acute kidney failure	5 (8.2)	9 (10.5)	0.82
Need for dialysis	4 (6.6)	5 (5.8)	>0.99
Need for postoperative pacemaker	5 (8.2)	12 (14.0)	0.28
Sepsis	30 (49.2)	23 (26.7)	**0.005**
Re-thoracotomy for bleeding	3 (4.9)	3 (3.5)	0.69
Postoperative CVA	5 (8.2)	2 (2.3)	0.13
Stroke	2 (3.3)	1 (1.2)	0.57
TIA	1 (1.6)	0	0.42
Seizure	2 (3.3)	1 (1.2)	0.57
Haemorrhagic transformation of preoperative stroke	1 (1.6)	0	0.42
Postoperative modified Rankin Scale	0 (0–1)	0 (0–2)	0.80
ICU stay (days)	3 (1–5)	3 (1–5)	0.48
Hospital stay (days)	15 (8–27)	16 (8-31)	0.63
In-hospital mortality	7 (11.5)	7 (8.1)	0.50
30-day mortality	13 (21.3)	13 (15.1)	0.37
Overall mortality	23 (37.7)	23 (26.7)	0.22

Data are presented as n (%) or median (25th–75th percentile). Bold values indicate statistically significant differences (*p* < 0.05). CVA: cerebrovascular accident; ICU: intensive care unit; TIA: transient ischemic attack.

## Data Availability

The data presented in this study are available on reasonable request from the corresponding author.

## Supplementary Materials

The following supporting information can be downloaded at: https://www.mdpi.com/article/10.3390/jcdd10080356/s1. Table S1: Demographic data of all 688 left-sided infective endocarditis patients with or without preoperative neurological complications, Table S2: Intraoperative data for early vs. delayed surgery after preoperative stroke, and Table S3: Multivariable logistic regression: Risk factors for postoperative neurological complications of early vs. delayed surgery after preoperative stroke (n = 147).
