# Supplementary material for: Early versus Delayed Surgery in Patients with Left-Sided Infective Endocarditis and Stroke
Source: J Cardiovasc Dev Dis. 2023 Aug 21;10(8):356. doi: 10.3390/jcdd10080356 (PMC10455129; doi:10.3390/jcdd10080356)
Supplement: Supplementary file 1 [file jcdd-10-00356-s001.zip › jcdd-2550493-supplementary.pdf]

Supplementary materials

Table S1. Demographic data of all 688 left-sided infective endocarditis patients with or without preoperative neurological complications

Variable	No preoperative neurological complications (n = 501)	Preoperative neurological complications (n = 187)	*p*-Value
Patient characteristics			
Age (years)	64 (52-72)	63 (55-71)	0.75
Female	114 (22.8)	61 (32.6)	**0.008**
BMI (kg/m²)	25.6 (23.5-29.2)	25.8 (23.7-29.3)	0.69
NYHA class			
I	20 (4.0)	5 (2.7)	0.41
II	87 (17.4)	19 (10.2)	**0.02**
III	233 (46.5)	88 (47.1)	0.90
IV	161 (32.1)	75 (40.1)	0.05
Coronary artery disease	276 (55.1)	96 (51.3)	0.38
Atrial fibrillation	102 (20.4)	49 (26.2)	0.10
Renal disease	194 (38.7)	75 (40.1)	0.74
Acute kidney failure	47 (9.4)	23 (12.3)	0.26
Dialysis	39 (7.8)	21 (11.2)	0.15
Hepatic disease	125 (25.0)	57 (30.5)	0.14
Previous stroke	58 (11.6)	33 (17.6)	**0.04**
Infective endocarditis characteristics			

Previous IE	25 (5.0)	13 (7.0)	0.32
Affected valve			
Aortic	343 (68.5)	109 (58.3)	**0.01**
Native	259 (51.7)	80 (42.8)	
Prosthetic	84 (16.8)	29 (15.5)	
Mitral	260 (51.9)	117 (62.6)	**0.01**
Native	222 (44.3)	106 (56.7)	
Prosthetic	38 (7.6)	11 (5.9)	
Double valve	102 (20.4)	39 (20.9)	0.89
Prosthetic valve endocarditis	96 (19.2)	36 (19.3)	0.98
Sepsis	148 (29.5)	86 (46.0)	**<0.001**
Congestive heart failure	179 (35.7)	55 (29.4)	0.12
Embolic events	66 (13.2)	165 (88.2)	**<0.001**
Brain	0 (0.0)	163 (87.2)	**<0.001**
Peripheral emboli	65 (13.0)	47 (25.1)	**<0.001**
Spleen	47 (9.4)	35 (18.7)	**0.001**
Lungs	3 (0.6)	4 (2.1)	0.09
Liver	2 (0.4)	4 (2.1)	0.05
Limbs	18 (3.6)	12 (6.4)	0.11
Kidney	12 (2.4)	7 (3.7)	0.34
Heart	3 (0.6)	5 (2.7)	**0.04**
Laboratory values			
C-reactive protein (mg/dL)	4.1 (1.6-9.4)	6.3 (2.5-13.1)	**0.001**
WBC (x10^9/L)	9.0 (7.1-12.3)	9.6 (7.5-14.0)	0.11
Creatinine (mg/dL)	1.1 (0.8-1.5)	1.0 (0.8-1.7)	0.42

Bilirubin (mg/dL)	0.6 (0.4-0.9)	0.6 (0.3-1.3)	0.64
Causative microorganism			
Pathogen isolated	351 (70.1)	143 (76.5)	0.10
Staphylococcus spp.	145 (28.9)	71 (38.0)	**0.02**
Staphylococcus aureus	89 (17.8)	62 (33.2)	**<0.001**
MRSA	12 (2.4)	5 (2.7)	0.79
Coagulase-negative staphylococci	56 (11.2)	9 (4.8)	**0.01**
Streptococcus spp.	101 (20.2)	45 (24.1)	0.27
Viridans group streptococci	70 (14.0)	24 (12.8)	0.70
Streptococcus bovis	22 (4.4)	14 (7.5)	0.11
Enterococcus spp.	73 (14.6)	13 (7.0)	**0.007**
HACEK group	3 (0.6)	1 (0.5)	>0.99
Fungi	1 (0.2)	1 (0.5)	0.47
Other	28 (5.6)	12 (6.4)	0.68
Annular abscess	158 (31.5)	74 (39.6)	0.05
Vegetation	374 (74.7)	155 (82.9)	0.26
Vegetation size (mm)	13 (9-17)	18 (13-24)	**<0.001**
Surgical characteristics			
Urgency of operation			
Elective	73 (14.6)	17 (9.1)	0.06
Urgent	250 (49.9)	82 (43.9)	0.16
Emergency	155 (30.9)	73 (39.0)	**0.045**
Salvage procedure	23 (4.6)	15 (8.0)	0.08
Bypass time (min)	135 (97-189)	147 (107-195)	0.14

Cross clamp time (min)	88 (63-121)	93 (69-122)	0.07
RBC (mL)	1225 (900-2100)	1800 (1200-2400)	**0.009**
FFP (mL)	0 (0-800)	400 (0-1000)	0.06
Reperfusion time (min)	32 (23-51)	35 (24-53)	0.17
Operation duration (min)	240 (186-328)	255 (191-329)	0.43
Site of valve surgery			
AV replacement	239 (47.7)	69 (36.9)	**0.01**
MV replacement	153 (30.5)	78 (41.7)	**0.006**
AV + MV replacement	98 (19.6)	34 (18.2)	0.68
AV replacement + MV repair	9 (1.8)	6 (3.2)	0.25
MV repair	2 (0.4)	0 (0.0)	>0.99
Type of valve surgery			
Biological prosthesis	218 (43.5)	92 (49.2)	0.18
Mechanical prosthesis	281 (56.1)	95 (50.8)	0.22
Concomitant surgery			
CABG	72 (14.4)	18 (9.6)	0.10
TV surgery	60 (12.0)	19 (10.2)	0.51
VSD repair	12 (2.4)	2 (1.1)	0.37
PFO repair	25 (5.0)	4 (2.1)	0.10
ASD repair	6 (1.2)	3 (1.6)	0.71
Aortic surgery	39 (7.8)	11 (5.9)	0.39
Splenectomy	18 (3.6)	1 (0.5)	**0.03**

Data are presented as n (%) or median (25th – 75th percentile).

Bold values indicate statistically significant differences ($p < 0.05$).

ASD: atrial septal defect; AV: aortic valve; BMI: body mass index; CABG: coronary artery bypass grafting; FFP: fresh frozen plasma; HACEK: *Haemophilus species, Aggregatibacter species,*

Cardiobacterium hominis, Eikenella corrodens, Kingella species; IE: infective endocarditis; MRSA: methicillin-resistant *Staphylococcus aureus*; MV: mitral valve; NYHA: New York Heart Association; PFO: patent foramen ovale; RBC: red blood cell; TV: tricuspid valve; VSD: ventricular septal defect; WBC: white blood cell

Table S2. Intraoperative data for early vs. delayed surgery after preoperative stroke

**Variable**	**Early surgery (n = 61)**	**Delayed surgery (n = 86)**	***p*-Value**
Time from stroke to surgery (days)	3 (1-6)	22 (13-36)	**<0.001**
Urgency of operation			
Elective	4 (6.6)	10 (11.6)	0.30
Urgent	23 (37.7)	42 (48.8)	0.18
Emergency	29 (47.5)	27 (31.4)	**0.047**
Salvage procedure	5 (8.2)	7 (8.1)	>0.99
Bypass time (min)	148 (100-187)	139 (111-196)	0.92
Cross clamp time (min)	95 (66-120)	91 (71-123)	0.95
RBC (mL)	1800 (1200-2400)	1500 (900-2175)	0.16
FFP (mL)	750 (0-1200)	0 (0-800)	**0.003**
Reperfusion time (min)	35 (23-54)	35 (25-49)	0.98
Operation duration (min)	245 (188-301)	255 (194-332)	0.58
Site of valve surgery			
AV replacement	20 (32.8)	35 (40.7)	0.33
MV replacement	28 (45.9)	35 (40.7)	0.53
AV + MV replacement	13 (21.3)	13 (15.1)	0.33
AV replacement + MV repair	0	3 (3.5)	0.27
MV repair	0	0	

Type of valve surgery			**0.04**
Biological prosthesis	25 (41.0)	50 (58.1)	
Mechanical prosthesis	36 (59.0)	36 (41.9)	
Size of implanted valve			
Aortic valve (mm)	23 (23-25)	23 (21-25)	0.07
Mitral valve (mm)	31 (29-31)	31 (29-31)	0.98
Concomitant surgery			
CABG	3 (4.9)	9 (10.5)	0.36
TV surgery	9 (14.8)	8 (9.3)	0.31
VSD repair	1 (1.6)	1 (1.2)	>0.99
PFO repair	1 (1.6)	2 (2.3)	>0.99
ASD repair	0	1 (1.2)	>0.99
Aortic surgery	1 (1.6)	7 (8.1)	0.14

Data are presented as n (%) or median (25th – 75th percentile).

Bold values indicate statistically significant differences ($p < 0.05$).

ASD: atrial septal defect; AV: aortic valve; CABG: coronary artery bypass grafting; FFP: fresh frozen plasma; MV: mitral valve; PFO: patent foramen ovale; RBC: red blood cell; TV: tricuspid valve; VSD: ventricular septal defect

Table S3. Multivariable logistic regression: Risk factors for postoperative neurological complications of early vs. delayed surgery after preoperative stroke (n = 147)

**Variable**	**OR**	**95% CI**	***p*-Value**
Enterococcus spp.	7.533	1.184-47.924	0.03
Operation duration (per 10 min increase)	1.059	1.002-1.119	0.04

CI: confidence interval; OR: odds ratio
